# High-dimensional hepatopath data analysis by machine learning for predicting HBV-related fibrosis

**DOI:** 10.1038/s41598-021-84556-4

**Published:** 2021-03-03

**Authors:** Xiangke Pu, Danni Deng, Chaoyi Chu, Tianle Zhou, Jianhong Liu

**Affiliations:** 1 Institute of Hepatology, The Third People’s Hospital of Changzhou, Changzhou, 213001 China; 2 School of Computer Science and Engineering, Jiangsu University of Technology, Changzhou, 213001 China; 3 Library, Jiangsu University of Technology, Changzhou, 213001 China; 4 Department of Neurosurgery, The First People’s Hospital of Changzhou, Changzhou, 213001 China

**Keywords:** Diseases, Mathematics and computing

## Abstract

Chronic HBV infection, the main cause of liver cirrhosis and hepatocellular carcinoma, has become a global health concern. Machine learning algorithms are particularly adept at analyzing medical phenomenon by capturing complex and nonlinear relationships in clinical data. Our study proposed a predictive model on the basis of 55 routine laboratory and clinical parameters by machine learning algorithms as a novel non-invasive method for liver fibrosis diagnosis. The model was further evaluated on the accuracy and rationality and proved to be highly accurate and efficient for the prediction of HBV-related fibrosis. In conclusion, we suggested a potential combination of high-dimensional clinical data and machine learning predictive algorithms for the liver fibrosis diagnosis.

## Introduction

Approximately 240 million individuals are infected with hepatitis B virus (HBV) worldwide, and roughly 600,000 of them died of HBV-related liver disease annually^1^. Chronic HBV infection, the main cause of cirrhosis and hepatocellular carcinoma, has become a global health concern^2^. Assessment of liver fibrosis status can assist doctors in determining the optimal timing and appropriate strategy for antiviral treatment to prevent disease progression of HBV-infected patients. Therefore, early diagnosis of liver fibrosis can benefit patients with HBV infection.

Currently, liver biopsy is the gold standard for fibrosis diagnosis, but its applicability is limited due to invasiveness, anesthetic complications, the risk of bleeding and sampling error. Non-invasive methods for stratifying fibrosis have been designed to overcome the inconveniences and disadvantages of liver biopsy^3^. Ultrasound elastography is an emerging non-invasive technology to assess liver fibrosis with good diagnostic accuracy. However, the stiffness measurements are affected by several factors such as liver vein congestion, cholestasis, inflammation, meal, obesity, ascites and observer experience, and finally result in elastography misinterpretation^4^. Hence, we are in bad need of an accurate and reliable non-invasive technology to diagnose liver fibrosis.

Machine learning algorithms are particularly adept at analyzing medical phenomena by capturing complex and nonlinear relationships in clinical data. Breast tumors can be quantitatively diagnosed based on subtle morphological variations of microenvironmental myoepithelial cells with 90.9% accuracy by a machine learning system^5^. Yip et al. developed a novel prediction model to detecting non-alcoholic fatty liver disease in the general population by machine learning algorithms on the basis of 23 routine laboratory and clinical attributes^6^. In an 800-person cohort, machine learning algorithms integrated dietary habits, blood markers, physical activities, anthropometrics and gut microbiota in order to accurately predict postprandial glycemic responses, and short-term personalized dietary interventions lowered postprandial glycemic responses successfully^7^. Although the usefulness of machine learning approaches has been proved in several medical fields, there is a stimulating debate on the accuracy, reliability and availability of machine learning algorithms in medical practice^8^.

In this paper, we devised a predictive model based on high-dimensional serum data to assess the liver fibrosis status among Asia HBV patients by machine learning algorithms and further evaluated the accuracy and rationality of the model. Here, we suggested a potential combination of high-dimensional clinical data and machine learning predictive algorithms for the liver fibrosis diagnosis.

## Material and methods

### Ethics statement

All methods were carried out in accordance with the Declaration of Helsinki, 2013. All experimental protocols were approved by the Ethics Committee of the Third People’s Hospital of Changzhou. Informed consent was obtained from all subjects or, if subjects are under 18, from a parent and/or legal guardian.

### Collection of HBV dataset

In this study, the data were obtained from 1023 patients with chronic hepatitis B at the Third People’s Hospital of Changzhou between 2015 and 2018. These patients have been confirmed to be HBV-infected by serological test, and the liver fibrosis status was assessed by biopsy. These patients were included according to the guidelines of prevention and treatment for chronic hepatitis B (2019 version)^9^. Patients with any other viral infections, or any other non-viral liver hepatitis were excluded. The average age of the patients was 55. Among these patients, 529 people were male, and 224 people were female. People who were infected with HBV genotype C accounted for 70% of these patients, and people with HBV genotype B accounted for 29%, and the proportion of the other different genotypes was less than 1%. These patients were divided into two groups: one was composed of patients with severe liver fibrosis (F4 according to the METAVIR scoring system) (F group)^10^, and the other was composed of patients with no or moderate fibrosis (F0-F3) (NF group). As the previous study has revealed, the data collected was consistent with the fact that HBV patients in East China with hepatitis B are almost genotype C and HBV genotype B^11,12^. Thus we focused on sample patients with genotype B and genotype C only.

### Description of the experimental attributes and units

On the purpose of making the experiment more rigorous and complete, we made diagnosis including all 55 biochemical attributes from the collected data. The descriptions of the 55 attributes were elaborated in our results. And we hypothesized that the 55 biochemical attributes were related to the two groups with different weights of influences.

### Naïve Bayes classifiers

The Naive Bayes classifier is considered as a common probabilistic classifier based on Bayesian statistics of strong independence assumptions. The above-mentioned two groups are defined as two classes: F and NF. The Naive Bayesian classification technique is used to develop the classifiers to discriminate between F and NF. In the vector F(*f*_1_,*f*_2_,…,*f*_*n*_) where *f*_1_,*f*_2_,…,*f*_*n*_ represent the biochemical attributes, each attribute estimates the probability distribution for the class F and NF. The Bayes’ theorem was described as:

$$P(c|F)=\frac{P(F|c)P(c)}{P(F)}$$

Here, the parameter c indicates the class variable (F or NF), *P*(*c*) is prior probability or marginal probability, *P*(*F*) is the constant for all classes, $P(F|c)$ and $P(c|F)$ denotes the posterior probability and conditional probability, respectively.

The Naïve Bayes classifiers in this study were developed by Rapidminer 8.0 (http://www.rapidminer.com/). In order to fit both the mixed and high-dimensional datasets, the Naive Bayes (Kernel) operator is chosen for its aptness in dealing with numerical attributes. The kernel in the operator is a weighting function for non-parametric estimations. What’s more, kernels are used to obtain random variables' density functions in kernel density estimation, thus providing an intuitive way to illustrate the computed results and making the whole process of machine learning interpretable and meaningful. The details of the experiment will be discussed later in the results.

### Parameter optimization and validating of the classification models

For the purpose of the optimal accuracy and appropriate computation complexity of the classifier, it is highly necessary to optimize the initial parameters and options.

First, the Laplace correction was applied in processing the data to prevent high influence of zero probabilities. The Laplace correction works by assuming that the training set is large enough so that adding a single data to each count will only make negligible variations in the estimated probabilities, but this could avoid the case of zero probability values. Then an option bandwidth was to be chosen through specifying a heuristic or fixed bandwidth. Note that the bandwidth of a kernel exerted a strong influence as a free parameter on the resulting estimate. Here an appropriate bandwidth was of great importance because the value will not be useful either if it is too small or too large, the same as the amount of kernels. In this study, a grid search was performed to obtain the optimal parameters as the number of which was limited. The both parameters of bandwidth and kernel were designed as values from 1 to 10, and they were both divided into 20 equal parts. After that, the two groups of parameters were cross-combined into 20 * 20-which is 400-combinations of parameters. For example, the two parameters can be combined as bandwidth is 1.5 and kernel is 2, or bandwidth is 7.5 while kernel is 5. The optimal pair of parameters was generated from the 400 combinations for the purpose of improving the accuracy of the prediction. Moreover, tenfold crossed validation was applied in the experiment. The dataset was divided into two parts, in which the 70% part is for training data and the 30% part is for testing data. Cross validation was applied to evaluate the prediction performance of the model and improve the performance of the new data in the trained model, which can reduce over-fitting to a certain extent. Besides, more effective information can be obtained from limited data as far as possible.

## Results

**Figure 1:**
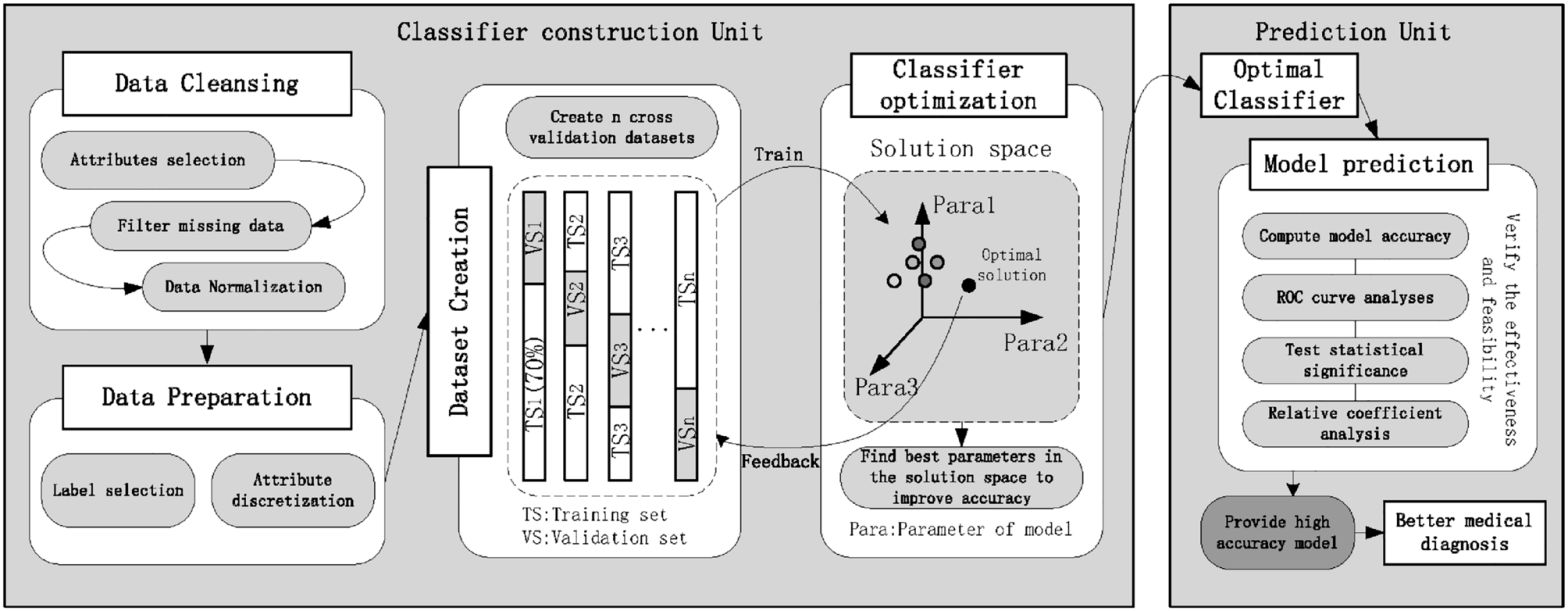
Diagram of fundamental steps in machine learning-based medical diagnosis construction.

To make the technological process observed intuitively, a flowchart was introduced to exhibit the whole experiment as Fig. 1.

### Pretreatment of the data

As is known that, problems with real-life data-such as data missing or disunity of value types-are unavoidable, usually making it unsuitable to input such data directly. For the sake of more accurate experimental results, we devoted much effort to the pretreatment of the raw data. First is data cleansing, targeting at the missing data and data in inappropriate value types. In this procedure, if the proportion of missing data exceeds 10%, this property is eliminated directly, and is excluded in the 55 properties. On the other hand, if the proportion of missing data is below 10%, the missing data is replaced with the average value of the whole property. As to the choice of a suitable data type, if there are only 2 types of attributes, the data type should be set to binominal, and multiple classes should be set to polynomial. The numeric attributes were converted into integer type. And other biological attributes such as ALT and AST were converted into numeric type. Besides, we also discretized numeric values in the data, as shown in Table S1. Therefore, not only the diagnosis accuracy was insured, but the process also made the calculation results more intuitive.

For example, as the original age data was discretized into polynomial type, the original interval [0, 80] was then divided equally into 3 equal parts as [0, 44], [45, 65] and [66, 80]. In this way, the difference between group F and group NF was able to be told easier. In addition, discretization also showed the ability in reducing computing complexity and preventing over-fitting. Accordingly, diagnosing the risk of fibrosis became faster when dealing with massive data. Moreover, statistical analysis for group F and group NF was conducted, including mean value, standard deviation and difference analysis. (Table 1 and Table S2).

**Table 1:** Statistical analysis of attributes between group F and group NF with significant difference.

Attributes	NF	F	p-value
Gender (male/female)	–	–	0
Age, years	43 ± 12	56 ± 11	0.02
ALT, U/L	73 ± 136	336 ± 439	0
AST, U/L	84 ± 290	177 ± 264	0.001
ALP, U/L	145 ± 152	112 ± 51	0.003
LDH-L, U/L	236 ± 154	184 ± 79	0.048
IDBIL, μmol/L	17 ± 15	13 ± 10	0.001
TP, g/L	66 ± 8	70 ± 6	0.007
ALB, g/L	36 ± 7	41 ± 4	0
GLO, g/L	29 ± 6	28 ± 5	0.004
A/G	1.3 ± 0.4	1.4 ± 0.3	0
CHOL, mmol/L	3.8 ± 1.1	3.8 ± 0.8	0.004
GLU, mmol/L	5.7 ± 1.9	5.4 ± 1.6	0.039
CRP, μmol/L	12 ± 24	4.3 ± 13	0
UREA, mmol/L	5.8 ± 3.8	4.1 ± 1.6	0.004
CREA,μmol/L	89 ± 54	77 ± 14	0.008
Ca, mmol/L	2.2 ± 0.2	2.1 ± 0.6	0
P, mmol/L	1.0 ± 0.2	1.0 ± 0.3	0.002
Mg, mmol/L	0.8 ± 0.1	0.7 ± 0.3	0
K, mmol/L	3.7 ± 0.5	3.4 ± 1.2	0
Na, mmol/L	138 ± 13	125 ± 42	0
Cl, mmol/L	102 ± 10	92 ± 31	0
TCO2, mmol/L	22 ± 3	20 ± 7	0
eGFR, ml/min	84 ± 24	98 ± 14	0
WBC, 10^9/L	4.9 ± 2.6	5.2 ± 1.7	0.007
EO%, %	2.4 ± 2.5	2.0 ± 1.6	0.014
BA%, %	0.60 ± 0.36	0.56 ± 0.29	0.027
NEUT#, 10^9/L	3.1 ± 2.3	3.0 ± 1.4	0.006
RBC, 10^12/L	4.1 ± 0.8	4.6 ± 0.7	0
HGB, g/L	127 ± 26	143 ± 23	0.002
HCT, %	37 ± 8	42 ± 7	0.003
MCV, fL	92 ± 7	91 ± 8	0.006
MCH, pg	31 ± 3.5	30 ± 3.3	0.009
PLT, 10^9/L	115 ± 74	158 ± 59	0.033
PCT, %	0.13 ± 0.08	0.17 ± 0.07	0.027
RDW-CV, %	14 ± 2.5	12 ± 1.6	0

### Result of ROC

As it is exhibited in Table 1, prevalence represents the proportion of positive samples, in which the fibrosis samples account for 84.7% of total, and the rest are negative samples. Generally speaking, the imbalance between the positive and negative samples will lead to the decrease of accuracy of data fitting and make it difficult to judge the quality of models. Therefore, an ROC curve was utilized to determine the prediction accuracy of diagnosing whether a sample is positive or not. Parameters for measuring the prediction performance of the models are shown in Table 2.

1. Positive Predictive Value (PPV) stands for the prediction accuracy of fibrosis (F) in fibrotic samples;
2. Negative Predictive Value (NPV) is the prediction accuracy of non-fibrosis (NF) in non-fibrotic samples;
3. True Positive Rate (TPR) represents the prediction accuracy of fibrosis in the whole sample;
4. Accuracy (ACC) shows the diagnosis accuracy in the whole sample;
5. Area under the ROC curve (AUC) is regarded as an index of judging whether the classifier is adequate for predicting both of the two samples (F&NF). The closer the value approaches 1, the better the classifier is.

**Table 2:** Predicting performance of the model.

Prevalence	Group	PPV (%)	NPV (%)	TPR (%)	TNR (%)	ACC (%)	AUC
84.70%	Modeling	99.53	86.52	97.25	97.47	97.29	0.982
	Test	99.29	78.95	95.42	96.15	95.53	0.972

Regarding to the table, the optimized naive Bayes model shows a good classification ability when predicting with positive samples. However, the success rate drops to 70–80% when predicting with the negative samples, in which the experiment is short of the negative cases also the fitting and learning procedure with negative sample data. Fortunately, the model still achieves an outstanding performance with a success rate of 95%. In addition, the modeling group in the table stands for the training dataset, which is at a proportion of 70% from the whole dataset.

It is clear that the success rate of test group is slightly lower than that of modeling group. Finally, with a tenfold cross-validation, the difference of accuracy between the two groups is limited, which indicates that the experiment method is conducive for handling nonlinear data fitting in machine learning.

### Analysis with Naïve Bayes model

1. ALT

**Figure 2:**
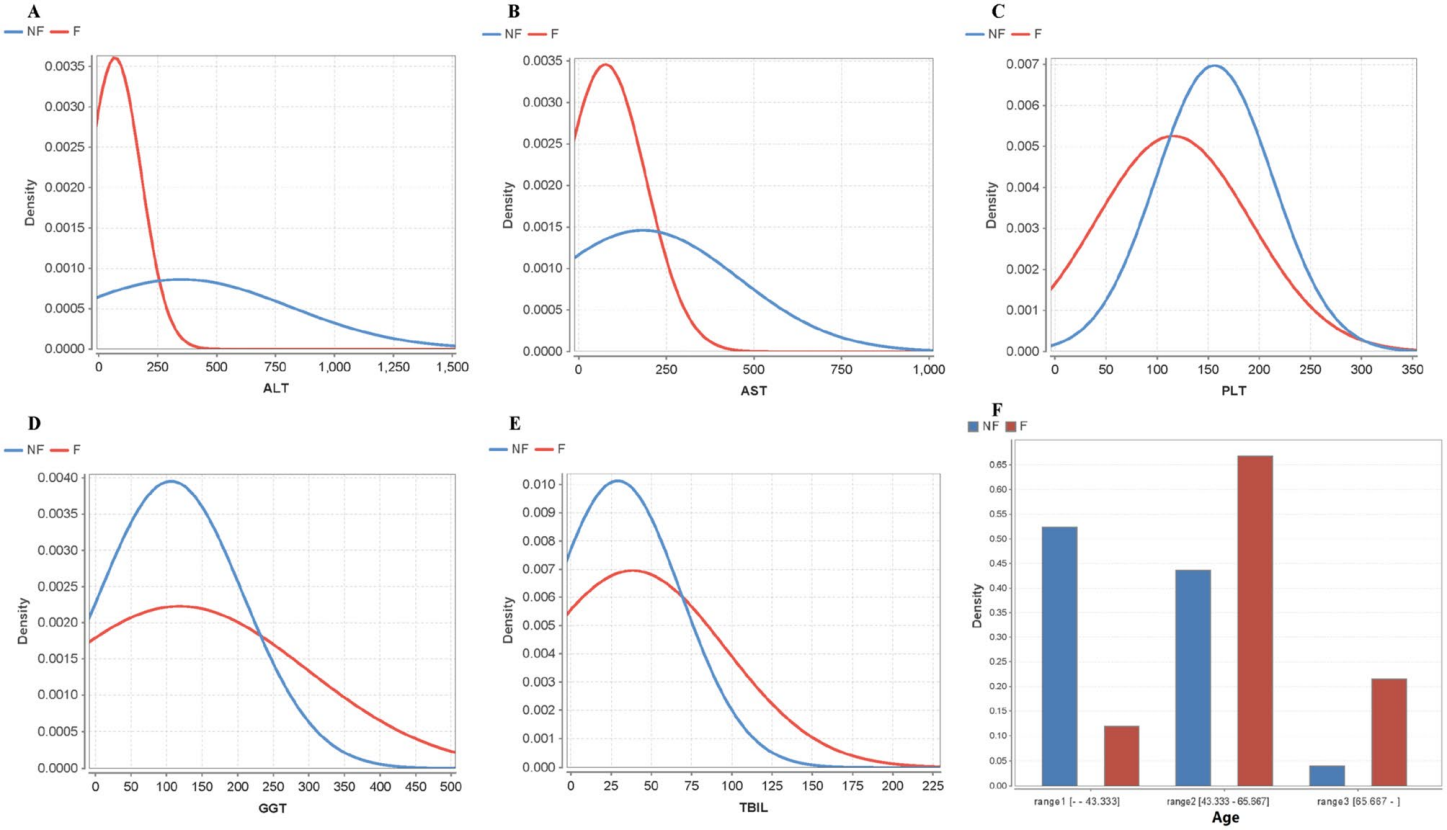
Effect to fibrosis of six main attributes by using optimized Naïve Bayes classifier. (**A**–**F**) The distribution of ALT, AST, PLT, GGT, TBIL and age related to fibrosis respectively.

By means of statistical analysis, the difference of ALT levels between NF group and F group is quite significant (p < 0.0001) and the average value of F group is 4.6(336/73) times of the average value of NF group (Fig. 2A). During the optimized modeling based on the Naïve Bayes algorithm, the probability distribution of F group and NF group obeys normal distribution when ALT varies from 0 to 1500, and when ALT is 50, the peak value of probability distribution of the F group is 5.17(0.0045/0.00087) times of the peak value of NF group. Besides, when ALT varies from 0 to 247, the probability of the F group is higher than the NF group, and when ALT is from 247 to 1500, the overall probability of NF group is higher than that of F group. The probability of NF group reaches the highest at 332, when the probability of NF group is 6.33(0.00095/0.00015) times of the probability of F group.

2. AST

In the statistical analysis, the difference of AST levels between NF group and F group shows high significance, and the average value of F group is 2.1(177/84) times of the NF group (Fig. 2B). After learning from the algorithm, AST level and ALT level also exhibit significant difference between F group and NF group. In the AST probability distribution between 0 and 249, the probability of F group is over all higher than the NF group probability, and the peak value of F group is 2.6 times (0.0035/0.00135) of the peak of NF group probability. In the rage from 249 to 1000, the probability of NF group is higher than that of F group, but the probability of both sides is decreasing.

3. PLT

The PLT levels of NF group and F group are significantly different (P < 0.05) based on the statistical analysis, and the F group average value is 1.37(158/115) times of the NF group average value (Fig. 2C). Via the learning procedure by the naive Bayes algorithm, it is clear to see that the NF probability distribution is better than the F probability distribution. When the value is in the interval from 110 to 272, the F probability is much higher than the NF probability, by which it means the increase in the number of platelets reduces the risk of fibrosis. In addition, the peak of F probability (platelets number at 155) is 1.5(0.0078/0.0052) times of the peak of NF probability (platelets number at 112), which means the risk of fibrosis is the lowest when the platelets number is around 155.

4. GGT

From the result of statistical analysis, the difference of GGT levels between NF group and F group is not statistically significant (P > 0.05). According to calculating results from machine learning, when GGT is in the interval from 0 to 230, the probability of NF group is much higher than that of the F group, and the probability of NF group to F group is at a ratio of 1.78 (0.004/0.00224) (Fig. 2D). For the interval from 230 to 500, the probability of the F group is higher than that of the NF group, and the weights of both the F group and the NF group decrease at the same time, which indicates that it is not worth paying attention to.

5. TBIL

Referring to the statistical analysis, the difference of TBIL levels between NF group and F group is not significant (P = 0.192). After calculated by machine learning, the result is as: when TBIL varies from 0 to 230, the probability of the F group is higher than that of the NF group and the ratio of the peak probability between the two groups is 1.07 (0.0077/0.0072) (Fig. 2E). The weight of the F group and the NF group decreases at the same time, and the proportion in the joint probability is not high enough, that is to say, the result of TBIL is of less significance.

6. Age

The result from the statistical analysis indicates that, the difference between the NF group and the F group is significant (p < 0.05), and the average value of the F group is 13(years) higher than that of the NF group (56–43) (Fig. 2F). Obviously, the risk of liver fibrosis is positively correlated with age. After analyzing with machine learning, a more intuitive result is given to describe the correlation between age and deterioration. First, the age attributes are discretized and divided into 3 parts, which can be labeled as 0–43, 43–65, 65 or older (years old). According to the graph, it is observed that the probability of the NF group is much higher than that of the F group during the age from 0 to 43, with a ratio of 4.5 (0.54/0.12). During the age from 43 to 65 years old, hepatitis B patients are nearly 62% (0.42/0.68) with fibrosis. After the age of 65, the ratio of the F group probability to the NF group probability is 5.25 (0.21/0.04), by which we can tell that only 19% of hepatitis B patients are fortunately exempted from fibrosis.

## Discussion

Our study proposed a predictive model on the basis of 55 routine laboratory and clinical parameters by machine learning algorithms as a novel non-invasive method for live fibrosis diagnosis. The predictive model is assessed on sensitivity, specificity, diagnostic efficiency, positive predictive value (PPV), negative predictive value (NPV), kappa value and area under the receiver-operating characteristic curve (AUC), and thus rivals with liver biopsy in accuracy.

Non-invasive predictive model using serum data is not new to liver fibrosis prediction, since the aspartate transaminase to platelet ratio index (APRI), the fibrosis index based on the four factors (FIB-4), the red cell distribution width-platelet ratio (RPR) and the gamma-glutamyl transpeptidase-to-platelet ratio (GPR) have been used for the detection of liver fibrosis and cirrhosis in medical practice for years^13–16^. These indexes usually make diagnosis based on at most 5 biological properties. In other words, these indexes assume that all the other attributes are independent or just weakly correlated to hepatic fibrosis. However, the sensitivity, specificity and efficiency of traditional regression algorithms are relatively poor compared with liver biopsy, due to limited dimension they can handle. On the purpose of making the experiment more rigorous and complete, we constructed a predictive model based on 55 routine laboratory and clinical parameters from a cohort of 1023 HBV patients, thus the accuracy of our model compared favorably with liver biopsy.

Machine learning, which is expert in analyzing high-dimensionally nonlinear data, is chosen to process the 55-dimensional data regression problem. However, the 55 dimensions of data were disunited in data types, which made even some machine learning model unable to handle with, such as SVM. Thus, a Naive Bayes algorithm was introduced to the experiment. As long as the parameters of the model are optimized, this algorithm is capable of gaining a high accuracy in non-linear data regression problem. It is well known that clinical data, such as AST, ALT, PLT, GGT and TBIL reflect the progression of liver fibrosis. Naive Bayes algorithm visualizes the relationship between clinical data and liver fibrosis for the first time, helping doctors further comprehend fibrosis progression.

More parameters should be included to further improve the predictive model, from personal information such as gender, body mass index, genomics, drinking and smoking habits, to disease history and medications associated with liver function, to novel data from emerging technologies such as transient elastography, computed tomography (CT) scan, MRI and virogene sequencing, to specific blood biomarkers related to fibrinogenesis or fibrinolysis, such as α-2 haptoglobin, macroglobulin, apolipoprotein A1, hyaluronate and tissue inhibitor of metalloproteinase I^17–19^. Besides, novel serum biomarkers for liver fibrosis attract our attention. YKL-40, an emerging inflammation biomarker, plays an important role in liver injury and fibrosis, thus can serve as a new fibrosis marker in chronic hepatitis B patients^20,21^. Glycosylated Wisteria floribunda agglutinin-positive Mac-2 binding protein (WFA + -M2BP), which is secreted from the liver cells during fibrosis progression, offers a new biomarker for fibrosis diagnosis among chronic hepatitis B patients^22,23^. Serum platelet-derived growth factor (PDGF) decreases remarkably as fibrosis progresses, thus can be used as a novel non-invasive biomarker for fibrosis assessment in chronic hepatitis B patients^24^. Serum miR-374, miR-29, miR-21, miR-223 and miR-143 levels vary with fibrosis progression, indicating that serum miRNA levels are potential noninvasive biomarkers of fibrosis progression^25,26^.

The greatest blemish of our research is the geographical limitation of hepatopath data. Hepatopath data in different regions, even different countries, should be included to extend the availability of the predictive model. What’s more, since the fibrosis progression is constantly evolving in response to new medications and technologies, database of patient information needs to be updated periodically. Subsequently, our model can automatically adjust to new data by adding new attributes and reassigning the weight for each attribute. Finally, clinical intervention may be adopted by targeting attributes with high weight to alleviate the fibrosis progression.

In conclusion, we constructed a valid, accurate and reliable prediction model for liver fibrosis and further detected characteristics from high-dimensional hepatopath data among Asia HBV patients with modern machine learning algorithms, suggesting the potential application of artificial intelligence to medical practice.

## Supplementary information

Supplementary tables.
