# Author Correction: High-dimensional hepatopath data analysis by machine learning for predicting HBV-related fibrosis

**DOI:** 10.1038/s41598-021-96032-0

**Published:** 2021-08-12

**Authors:** Xiangke Pu, Danni Deng, Chaoyi Chu, Tianle Zhou, Jianhong Liu

**Affiliations:** 1 Institute of Hepatology, The Third People’s Hospital of Changzhou, Changzhou, 213001 China; 2 School of Computer Science and Engineering, Jiangsu University of Technology, Changzhou, 213001 China; 3 Library, Jiangsu University of Technology, Changzhou, 213001 China; 4 Department of Neurosurgery, The First People’s Hospital of Changzhou, Changzhou, 213001 China

Correction to: *Scientific Reports* 10.1038/s41598-021-84556-4, published online 03 March 2021

The original version of this Article contained an error in Affiliation 2 and Affiliation 3, where the city was incorrectly given as ‘Zhenjiang’. The correct affiliations are listed below.

School of Computer Science and Engineering, Jiangsu University of Technology, Changzhou 213001, China.

Library, Jiangsu University of Technology, Changzhou 213001, China.

The original Article has been corrected.

