# Supplementary material for: High-dimensional hepatopath data analysis by machine learning for predicting HBV-related fibrosis
Source: Sci Rep. 2021 Mar 3;11:5081. doi: 10.1038/s41598-021-84556-4 (PMC7930086; doi:10.1038/s41598-021-84556-4)
Supplement: Supplementary file 1 — Supplementary tables. [file 41598_2021_84556_MOESM1_ESM.docx]

**Table S1. Discretization for values**

| Attributes | Group1 | Group2 | Group3 |
| --- | --- | --- | --- |
| Age, years | 0-43 | 43-65 | 65- |
| RDW, % | 10.5-12.5 | 12.5-14.5 | 14.5-18 |
| MPV, fL | 10.5-11 | 11-12.5 | 12.5-14 |
| PDW, % | 0-14 | 14-19 | - |
| NEUT#, 10^9/L | 0-3 | 3-7 | - |
| BA%, % | 0-1 | 1-2 | - |
| EO%, % | 0-2 | 2-5 | - |
| WBC, 10^9/L | 30-40 | 40-50 | 50-60 |
| Na, mmol/L | 0-146 | 146- | - |
| GLU, mmol/L | 3-5 | 5-10 | - |
| CHOL, mmol/L | 0-3 | 3-7 | - |

**Table S2. Statistical Analysis of Attributes between Group F and Group NF without Significant Difference**

| Attributes | NF | F | p-value |
| --- | --- | --- | --- |
| GGT, U/L | 113±164 | 105±99 | 0.07 |
| ADA, U/L | 22±11 | 18±11 | 0.121 |
| TBIL, μmol/L | 37±56 | 30±46 | 0.192 |
| DBIL, μmol/L | 20±43 | 17±35 | 0.523 |
| PA, mg/L | 14±7 | 7±8 | 0.483 |
| TBA, μmol/L | 38±53 | 29±57 | 0.519 |
| CHE, U/L | 5083±2556 | 6096±3070 | 0.152 |
| TG, mmol/L | 1±0.6 | 1.2±0.6 | 0.509 |
| UA, μmol/L | 302±112 | 266±119 | 0.322 |
| NEUT%, % | 61±12 | 55±12 | 0.466 |
| LY%, % | 26±11 | 31±10 | 0.172 |
| MO %, % | 9.3±3.1 | 9.3±2.7 | 0.465 |
| LY#, 10^9/L | 1.2±0.6 | 1.6±0.6 | 0.864 |
| MO#, 10^9/L | 0.45±0.26 | 0.50±0.21 | 0.114 |
| EO#, 10^9/L | 0.11±0.12 | 0.11±0.09 | 0.075 |
| BA#, 10^9/L | 0.015±0.036 | 0.018±0.038 | 0.079 |
| MCHC, g/L | 340±17 | 335±29 | 0.98 |
| PDW, % | 13±4.5 | 13±4 | 0.214 |
| MPV, fL | 10±3 | 10±2.5 | 0.118 |
